# Practitioners' Bookshelf

**Published:** 1892-06-25

**Authors:** 


### PRACTITIONERS' BOOKSHELF.

### A MANUAL OF CHEMISTRY.*

This work is intended chiefly for the use of students of medicine, and we cordially endorse the hope expressed by the author that it will supply a long-felt want. The study of chemistry has hitherto always been a matter of difficulty to the student of medicine, owing to the fact that, on the one hand, he has had no means of knowing the amount and kind of knowledge required of him, and, on the other hand, that he had been obliged to obtain his information from so many different sources. The book before us, however, has been designed especially with the object of meeting and overcoming these difficulties, and the student of medicine will now be enabled to obtain, in a concise and reliable form, the necessary knowledge in this branch of his studies. The ground covered will fully satisfy all the requirements of the different examinations which the ordinary student of medicine is called upon to undergo, and the book will probably be found of use later as a means of reference to those portions of chemical science which bear on the study and practice of medicine.

The book is divided into five parts. The first seventy pages, comprising Part I., contain an excellent introduction to the study of chemistry, and may be read with profit, not only by the student of medicine, but also by the student of chemistry. This part, indeed, is one of the most interesting to be found in the whole book, and the author is to be congratulated on the clear and concise manner in which he has treated a part of his subject which bristles with difficulties for beginners. Part II. deals with "The non-metallic elements, and their principal compounds." This part, as well as Part III., on "The metallic elements and their principal compounds," does not call for detailed criticism. The arrangement of the different chapters is good, and the headings at the beginning of each chapter are especially worthy of commendation. The various experiments described are carefully chosen, and the different reactions obtained are admirably explained and illustrated, and we observe with pleasure that a praiseworthy economy has been exercised in the matter of engravings, which, on the whole, are good. Part IV. treats of organic chemistry. Contrary to our expectations, we do not think that this portion of the book is so good as that which precedes it. The subject matter of most of the chapters in this part is too compressed, and in some places lucidity has been sacrificed to brevity. This observation applies especially to chapters IV., IX., X., XI., which would all be greatly improved by judicious expansion, and we hope this will be done in future editions of the work. Apart from this blemish, the work in this section is of a very high order. Part V. consists of chemical problems, and forms a most useful addition to the book. The different problems are carefully chosen, and the methods of answering them are clearly and accurately described, and they should be of great assistance to the student as a means of testing his knowledge of his subject. Directions for examining a salt or solution containing not more than one metal and one acid, which are arranged in tabular form, complete this section. The book is well printed, and, so far as we have been able to discover, contains a full and reliable index.

We can confidently recommend this "Manual of Chemistry" to students of medicine, and we have little doubt that it will deservedly become a popular work in this branch of their studies.

* "A Manual of Chemistry (Inorganic and Organic)." By Arthur P. Luff, M.D., B.Sc. (Lond.), M.R.C.P. (London, Paris, and Melbourne: Cassell and Co. 1892.)

### CATECHISM SERIES.—ANATOMY.*

These little books are fairly correct as far as we have looked into them, but we in no way advise the medical student to rely on his "Catechism Series" to carry him through *any* anatomy examination without the aid of some of the standard works on anatomy, and diligent study in the dissecting-room.

* E. and S. Livingstone, Edinburgh.

